# Precision of post-operative localization of deep brain stimulation electrodes

**DOI:** 10.1038/s41598-025-01449-6

**Published:** 2025-05-28

**Authors:** Andrej Lasica, Pavel Filip, Kristína Burdová, Josef Mana, Filip Růžička, Dušan Urgošík, Karsten Mueller, Dimitra Kiakou, Robert Jech

**Affiliations:** 1 Department of Neurology, First Faculty of Medicine, General University Hospital in Prague, Charles University, Kateřinská 30, 120 00 Prague, Czech Republic; 2 Center for Magnetic Resonance Research (CMRR), University of Minnesota, Minneapolis, MN USA; 3 Department of Radiology, Na Homolce Hospital, Prague, Czech Republic; 4 Max Planck Institute for Human Cognitive and Brain Sciences, Leipzig, Germany

**Keywords:** Deep brain stimulation, Parkinson’s disease, Neuroimaging, Magnetic resonance imaging, Subthalamic nucleus, Electrode localization, Software comparison, Neuroscience, Neurology, Neurological disorders

## Abstract

Tools for post-operative localization of deep brain stimulation (DBS) electrodes may be of major benefit in the evaluation of the stimulation area. However, little is known about their precision. This study compares 3 different software packages used for DBS electrode localization. T1-weighted MRI images before and after the implantation of the electrodes into the subthalamic nucleus for DBS in 105 Parkinson’s disease patients were processed using the pipelines implemented in Lead-DBS, SureTune4, and Brainlab. Euclidean distance between active contacts determined by individual software packages and in repeated processing by the same and by a different operator was calculated. Furthermore, Dice coefficient for overlap of volume of tissue activated (VTA) was determined for Lead-DBS. Medians of Euclidean distances between estimated active contact locations in inter-software package comparison ranged between 1.5 mm and 2 mm. Euclidean distances in within-software package intra- and inter-rater assessments were 0.6–1 mm and 1–1.7 mm, respectively. Median intra- and inter-rater Dice coefficients for VTAs were 0.78 and 0.75, respectively. Since the median distances are close to the size of the target nucleus, any clinical use should be preceded by careful review of the outputs.

**Supplementary Information:**

The online version contains supplementary material available at 10.1038/s41598-025-01449-6.

## Introduction

Deep brain stimulation (DBS) is an established treatment of various neuropsychiatric disorders, most commonly Parkinson’s disease, dystonia, and essential tremor. Various DBS targets are utilized depending on the diagnosis. For Parkinson’s disease, DBS is implanted in the subthalamic nucleus (STN); the globus pallidus internus is utilized in dystonia, and the ventral intermediate nucleus of the thalamus in essential tremor^1^. The correct final position of the implanted electrode is essential for successful therapy. Extensive preoperative preparation is therefore required, including the acquisition of various structural Magnetic Resonance Imaging (MRI) images to precisely locate the target structure and insertion trajectory. Despite the many difficulties associated with planning and the surgical procedure itself, a mere “hit” of the target structure may not be fully sufficient to elicit the coveted clinical effects. Different parts of the nuclei can be associated with a different profile of adverse effects, efficacy, and therapeutic window^2–4^.

Consequently, multiple tools were developed for post-operative localization of the DBS leads and the estimation of the volume of tissue activated (VTA) based on the stimulation position and settings. Currently, there is no universally accepted tool and researchers generally choose packages based on their research needs. One of the best known options is a MATLAB toolbox called Lead-DBS (https://www.lead-dbs.org)^5^ available as a freeware. Furthermore, there are commercial software packages, like SureTune4™ (version 4, 2023) (Medtronic, Minneapolis, MN, USA), BrainLab Elements™ (Brainlab AG, Munich, BY, DE). Although Lead-DBS is compatible with all the commonly used leads, SureTune4™ supports only Medtronic electrodes and BrainLab Elements™ has a library for Boston Scientific electrodes only. However, BrainLab Elements™ allows for customization of other electrode models.

While an ultimate evaluation of the accuracy of individual software packages would require histological exploration of the brain post-mortem, the delineation of their precision, i.e. the ability to produce replicable results, is a more manageable and implementable approach. Hence, the presented study aimed to assess the similarity of results of individual software packages developed for the localization of DBS electrodes in the native space of the patient. Furthermore, within-software package variability was evaluated when the processing pipeline was repeated by the same (intra-rater variability) and by another operator (inter-rater variability). We hypothesized that all these approaches will yield similar results well below the size of the target structure of interest – STN, with its average width of about 6 mm^6,7^.

## Results

3 subjects were excluded from SureTune4™ pipeline due to insufficient quality of MRI images and 3 subjects were excluded from BrainLab Elements™ pipeline due to the incompatibility of MRI data with BrainLab Elements™ (non-amendable failure to load the scans). Ergo, the final analysis is based on 99, 101, and 103 subjects, for Brainlab vs. SureTune4, Brainlab vs. Lead DBS, Lead-DBS vs. Brainlab respectively.

Basic demographic, clinical and DBS-related information (hardware and stimulation settings) are provided in Table 1.

**Table 1:** Basic demographic, clinical and DBS-related information (hardware and stimulation settings).

		Count	
**Age (years)**	58.5 [8.25]	105	
**Disease duration (years)**	14.68 [5.9]	98	
**Sex (count of F/M)**	37/68	105	
**Time since disease onset (years)**	2.47 [2.88]	105	
**DBS-related information**			
Time since DBS implantation (months)	29.68 [34.57]	105	
Stimulator type [Infinity / Kinetra / Activa / Percept]	40 / 3 / 56 / 6	105	
Electrode type [3389 / B33005 / 6172 / 6170 / 6171 / 6178]	64 / 2 / 24 / 13 / 1 / 1	105	
Stimulation mode [monopolar / bipolar / interleaved]	102 / 2 /1	105	
Constant voltage / constant current mode Voltage amplitude (V) (bilat. average) Current (mA) (bilat. average)	33 / 72 3.325 [0.48] 2.2 [0.66]	105	
Pulse width (µs)	65.07 [11.15]	101	
Frequency (Hz)	129.21 [9.98]	101	
Total electrical energy delivered (µW)	53.12 [44.74]	79	
Impedance (Ω)	1166 [342.62]	82	

Data is provided as an average [ standard deviation].

DBS – deep brain stimulation; STN – subthalamic nucleus.

**Fig. 1:**
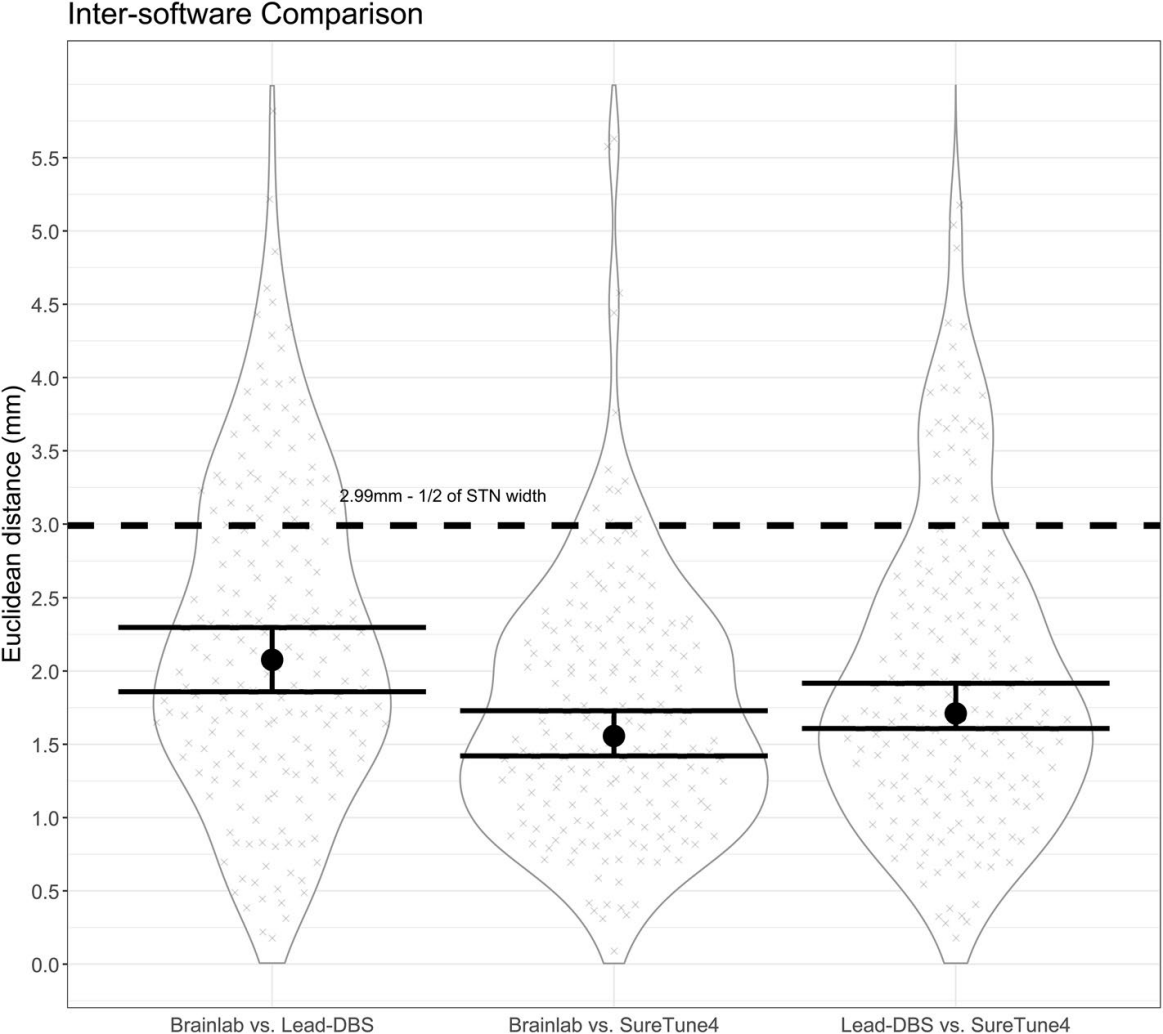
Violin graphs for inter-software package comparisons. Medians of Euclidean distances between positions of active contacts estimated by individual software packages are marked with a black dot, with 95% confidence intervals shown with the error bars. Dashed black line marks the width of the subthalamic nucleus for reference as the predetermined threshold of clinical significance. BrainLab Elements was shortened to BrainLab due to space reasons.

Median Euclidean distances between the locations of active contacts estimated by individual software packages were in the range of 1.56–2.08 mm (6–26% of subjects with distances over 2.99 mm), with the highest agreement found between BrainLab Elements™ and SureTune4™ and the lowest agreement was observed between BrainLab Elements™ and Lead-DBS. These distances were equivalent under the predetermined conditions (1/2 the width of the STN was considered as a clinically relevant threshold) (*p* < 0.001 after FDR correction). For full results, see Table 2; Fig. 1.

**Table 2:** Inter-software package comparison – Euclidean distances between positions of active contacts estimated by individual software packages, differences between Polar angles Θ and Φ, presented as medians (10th − 90th percentile).

Compared software packages	Euclidean distance [mm]	θ [°]	φ [°]	Cases over the threshold [%]	*P* value (FDR)
BrainLab Elements vs. Lead-DBS	2.076 (0.829–3.63)	21.47 (4.75–46.11)	10.51 (2.96–19.54)	25.88%	< 0.001
BrainLab Elements vs. SureTune4	1.557 (0.793–2.71)	21.37 (5.94–47.21)	12.96 (4.9–22.7)	6.21%	< 0.001
SureTune4 vs. Lead-DBS	1.710 (0.818–3.585)	6.21 (1.18–17.29)	4.57 (1.18–12.73)	15.27%	< 0.001

Furthermore, the percentage of subjects with distances over the predetermined threshold of 2.99 mm - 1/2 of the width of the subthalamic nucleus is presented. P values after FDR correction of equivalence tests are provided. Abbreviations: FDR – false discovery rate.

**Fig. 2:**
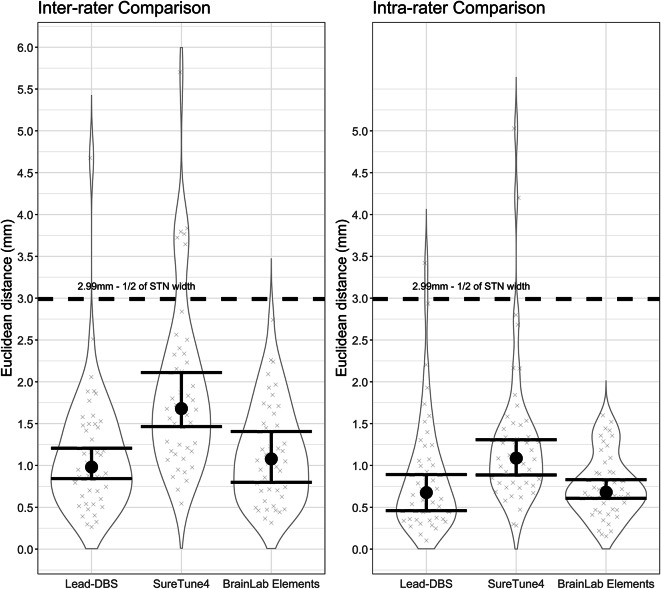
Violin graphs for within-software package inter- (**A**) and intra-rater (**B**) comparisons separately in individual software packages. Medians of Euclidean distances between positions of active contacts estimated by individual software packages are marked with a black dot, with 95% confidence intervals shown with the error bars. The dashed black line marks the width of the subthalamic nucleus for reference as the predetermined threshold of clinical significance.

Within-software package intra-rater variability analysis yielded Euclidean distances of 0.68 mm (BrainLab Elements™), 0.676 (Lead-DBS), and 1.08 mm (SureTune4™), while within-software package inter-rater Euclidean distances 1.07 mm (BrainLab Elements™), 0.981 (Lead-DBS), and 1.68 mm (SureTune4™). For full results, see Table 3; Fig. 2. Lastly, Dice coefficients for Lead-DBS VTA overlap for intra-rater, and inter-rater comparison were 0.778 (0.563–0.877), and 0.748 (0.488–0.869), respectively (medians [10th and 90th percentile]).

**Table 3:** Within-software package intra- and inter-rater comparisons – Euclidean distances between the estimated positions of the active contacts, differences between Polar angles Θ and Φ.

Software	Lead - DBS		SureTune4		BrainLab Elements	
Rater	Same	Different	Same	Different	Same	Different
Euclidean Distance [mm]	0.676 (0.299–1.63)	0.981 (0.455–1.86)	1.08 (0.063–2.16)	1.68 (0.942–3.66)	0.681 (0.331–1.35)	1.07 (0.47-2.0)
θ [°]	1.26 (0.31–4.71)	2.39 (0.61–5.69)	1.64 (0.24–4.85)	2.74 (0.36–7.65)	1.39 (0.40–4.01)	1.83 (0.28–7.41)
φ [°]	0.97 (0.12–2.26)	1.39 (0.42–3.35)	1.03 (0.29–2.57)	3.32 (0.87–8.29)	0.7 (0.13–1.75)	0.63 (0.16–2.28)
Cases over the threshold [%]	2%	2%	4%	15%	0%	2%
p value (FDR corrected)	< 0.001	< 0.001	< 0.001	< 0.001	< 0.001	< 0.001

Presented as medians (10th − 90th percentile). Furthermore, the percentage of subjects with distances over the predetermined threshold of 2.99 mm - 1/2 of the width of the subthalamic nucleus is presented. P values after FDR correction of equivalence tests are provided.

FDR – false discovery rate.

Additionally, analysis of Euclidean distances, and polar angles of active contacts, and electrode trajectories after reversion of coordinate transformations from BrainLab Elements and Lead-DBS is provided in supplementary materials (Supplementary Figs. 3 and 4, supplementary Table 4), together with analysis of axial differences (supplementary Fig. 5), and analysis of precision based of the closest distance from Bejjani line^8^ (Supplementary Figs. 6 and 7).

## Discussion

Software packages designed to localize the DBS electrodes are becoming an important part of DBS programming and DBS research. For the time being, the processing pipelines contain multiple manual steps, where the experience of the rater is important not only to delineate the precise location of the lead in the MRI artifact area, but also to evaluate and optimize the accuracy of image registration. Moreover, the size of STN when combined with the resolution of structural MRI scans, presents a significant challenge in the process. Nevertheless, studies aimed at the precision of utilized software package are lacking and generally limited to MNI space^9^, phantom studies^10^, or contain a low numbers of subjects without analysing the repeatability of the process within one software^11^.

The process of DBS lead localization shares several steps in all the software packages considered in this study. Preop T1w scans and postop T1w or CT scans are generally needed. The first step is the co-registration of the postop scan to the preop image. Co-registration in SureTune4™ is manual, BrainLab Elements™ uses an automated, but not disclosed algorithm, and Lead-DBS offers multiple approaches, the default being a rigid-body transformation derived from SPM12^12^. In Lead-DBS, the basic co-registration is improved by brainshift correction using threefold linear registration^13^. This step may have been one of the plausible factors behind the lower concordance of Lead-DBS results with the two other software packages. The final step is the DBS electrode localization. SureTune4™ and BrainLab Elements™ use manual localization of the lead. Lead-DBS uses semi-automated TRAC-CORE^14^ for postop T1w and eventually CT acquisitions, also offering the supposedly superior automated PACER algorithm^15^ for CT scans only. This Lead-DBS step is then followed by manual fine adjustment. Overall, electrode localization is the most important step in the pipeline and possibly the main source of error. And lastly, while not immediately evident if working only in one software package, BrainLab Elements™ and SureTune4™ operate in the AC/PC coordinate system, with BrainLab Elements™ transforming the images in a way that the reference point (point 0,0,0) is MCP, the midline of the brain coincides with z axis and AC/PC line coincides with the y axis. This transformation may well have been the reason behind the difference of BrainLab Elements™ polar angles from other software packages.

Our results show a generally good agreement of the software packages considered. Nevertheless, the observed agreement is uncomfortably close to the predetermined clinically relevant threshold of 1/2 width of the STN (2.99 mm), which can also mean that the selected threshold was too high. Should the distance between lead contacts (2 mm between the centres of adjacent contacts or even 0.5 mm as the inactive gap between two adjacent contacts) be utilized as the clinically relevant threshold, the employed equivalence tests would fail. Since the utilization of another DBS contact may yield substantially different clinical results, this more stringent threshold is definitely a viable option as well. Nevertheless, when software-based localization is used for programming, the results are comparable with the current gold standard of care^16–19^. Unsurprisingly, the software package utilizing the least automated pipeline (SureTune4™) numerically provided the highest inter- and intra-rater variability, while Lead-DBS and BrainLab Elements™ were closely matched in their performance. The inter-software package comparison yielded generally similar results for all the packages. While no definitive verdict may be provided on the accuracy of individual tools, their precision may be considered equivalent under the utilized assumptions. However, considering the structural and functional heterogeneity of STN subsections^20^, it remains to be seen whether it’s sufficient for clinical applications. Also, intra- and inter-rater Lead-DBS VTA Dice coefficient at the level of 0.778 and 0.748, respectively, may be considered surprisingly low, considering that the VTAs were created by the same algorithm and the only difference was the (sub millimetre) distance offset. Lead-DBS uses a finite elements method to calculate the VTA. This unfortunately means that repeated estimation of VTA using this software will yield slightly different results. Hence, the calculation method and the difference in the position are the main contributors of this rather sizeable difference. This mediocre similarity puts into question the utility of this tool for clinical practice.

And lastly, when comparing the polar angles, polar angles derived from BL differ substantially from the two other compared software packages. However, polar angles after reversion of transformation generally matched, which means that the polar angle differences were mostly because of the software transformations (Supplementary Table 4).

There are several limitations to be considered in this study. First, we used post-operative MRI images for electrode localization rather than CT scans – a procedure more common in the clinical practice. Moreover, the comparison of CT and MRI localizations has been reported to provide equal outcomes^21^. Second, standard transformations limited to only select software packages (brain shift correction in Lead-DBS and the AC/PC transformation in BT) were not reverted in the main analysis, since they are a part of standard respective pipelines, however analysis after reversion is provided in supplementary materials for comparison (Supplementary Figs. 3 and 4, Supplementary Table 4). Third, the dataset was rather diverse, including subjects with various disease durations, times since the implantation, and acquired at different MRI systems using different MRI protocols. Although all the calculated measures were within-subject comparisons, the performance of the considered software packages may differ in more substantial disease-related atrophy and/or T1w signal intensity changes. However, this diverse dataset generally corresponds to the conditions seen in clinical practice. And finally, Lead-DBS is the only software package considered in this paper which provides information about the electrode position with respect to the STN. Therefore, it was not possible to calculate any relevant comparison of within-STN VTAs – a metric possibly associable with the clinical effectiveness of STN DBS. Hence, the qualitative visual output of software packages showing the positions of individual contacts in the STN, even though putatively informative for the neurologist adjusting the stimulation parameters in the clinical settings, must be interpreted with caution given the clinically non-negligible positional differences between the software packages. The output of multiple tools must be evaluated, should these visualizations be considered in the clinical settings before further tools enable the export of within-STN VTAs and/or STN masks for further comparisons and correlations with clinical outcomes.

In conclusion even though the performance of SureTune4™, BL and Lead-DBS may be considered equivalent under conditions adopted in this study, the Euclidean distances between the estimated positions of active contacts are rather close to 2.99 mm and generally exceed the distance between two neighbouring contacts of the lead. Clinicians and researchers should exercise utmost care in the implementation of the estimated locations of contacts in their respective fields.

## Methods

### Subjects

105 patients with idiopathic Parkinson’s disease (PD) with chronic bilateral STN DBS were included in this study. Further inclusion criteria were as follows: available pre-operational T1-weighted (preop T1w) scan, bilateral STN DBS implantation and T1w acquisition at least 3 months after DBS implantation (postop T1w). Implanted hardware specifications and stimulation parameters used at the time of this second MRI acquisition were recorded.

The study protocol was approved by the Ethics Committee of the General University Hospital in Prague and every subject signed a written informed consent in accordance with the Declaration of Helsinki.

### Imaging protocol

Since the subjects included in this study underwent DBS implantation and the preoperative MRI acquisition over a large time span (from 2008 to 2022), several different MRI systems and acquisition protocols were used The most common imaging parameters were: pre-operative scans were acquired using a Siemens Skyra system (Siemens, Erlangen, Germany): (1) T1-weighted MPRAGE images: In-plane resolution 1 × 1 mm^2^, Slice thickness 1 mm, 1 mm interslice gap, 176 slices, TR 2200ms, TE 2.43ms FA 8°. (2) T2-weighted Spin-Echo sequence: In-plane resolution 0.94 × 0.94mm^2^, Slice thickness 2 mm, 2 mm interslice gap, 28 slices, TR 2440ms, TE 80ms, FA 90°. Post-operative scans were acquired using a Siemens Symphony system (Siemens, Erlangen, Germany): T1-weighted MPRAGE images: In-plane resolution 0.45 × 0.45mm^2^, Slice thickness 0.9 mm, 0.9 mm interslice gap, 176 slices, TR 2060ms, TE 3.93, FA 15°. Exact imaging parameters are presented in Supplementary Tables 1, 2 and 3.

### Lead-DBS pipeline

Standard Lead-DBS^5^ (version 2.5.3) on MATLAB (version 2021b) (Mathworks, Natick, MA, USA) pipeline consisted of preprocessing, electrode localization, and VTA estimation. Postop T1w scans were coregistered to preop T1w scans using diffeomorphic registration as implemented in SPM12 ^12^. This approach failed in 18 subjects, where the Advanced Normalization Tools (ANTs; http://stnava.github.io/ANTs/)^23^ package was used. Afterwards, images were spatially normalized to MNI space using ANTs. The brain shift correction was performed via 3-fold linear registration^13^. The localization of the electrode included 2 steps. First rough localization was based on the semi-automated TRAC-CORE algorithm^14^, which was then finalized manually in Lead-DBS graphic user interface. VTA estimation was based on the FieldTrip-SimBio algorithm with a white matter conductivity set to 0.14 S/m, grey matter conductivity set to 0.33 S/m (default settings), and subsequent magnitude thresholding of the electric field gradient at the level of 0.2 V/mm^24^. Since SureTune4™ and BrainLab Elemenets™ do not support segmented contacts, their coordinates were converted to simple ring contacts as centres of circles defined by the 3 individual contacts. And lastly, the coordinates of the active contact and VTAs were transformed to the subject’s native space and exported, with mid-commissural point (MCP) manually set as the zero point of the coordinate system.

### SureTune4 pipeline

Manual registration of the postop T1w acquisition to the preop T1w image and the Anterior commissure – Posterior commissure (AC-PC) line was determined. Similar to Lead-DBS, the first rough manual electrode localization was followed by fine electrode adjustment. Since SureTune4™ does not support St. Jude electrodes, all electrodes were modelled as Medtronic electrode 3389 (equivalent electrode size, contact length and spacing). The coordinate system was transformed from the Left-Posterior-Superior to the Right-Anterior-Superior definition to ensure the compatibility with the Lead-DBS output. In the last step, the coordinates of the active contact in the subject’s native space (with MCP manually set as the zero point) were exported.

### Brainlab pipeline

The semi-automated registration of the postop T1w image to the preop T1w image with manual optimization was performed in BL (image fusion version 3.0.1.6 RELEASE) graphic user interface and the AC-PC line was determined. The mid-commissural point (MCP) was set as a reference point and the manual 2-step localization (version 2.5.1.5 RELEASE) of the DBS lead was performed. The polar coordinates (θ and φ) were calculated from the Cartesian coordinates of the lead target and lead entry point and the positions of individual contacts were calculated based on θ and φ (for visual explanation of this process, see the Supplementary Fig. 1). Afterwards, the coordinate system was transformed from the Left-Posterior-Superior to the Right-Anterior-Superior definition and the coordinates of the active contact in the subject’s native space (with MCP manually set as the zero point) were exported.

### Comparison of active contact position and VTA overlap

Further analysis involved the calculation of the Euclidean distance between the positions of active contacts as determined in each software package. Additionally, differences of absolute values of θ φ angles were calculated.

To delineate the intra- and inter-rater variability, 25 subjects, chosen randomly from the whole dataset, were processed again in all 3 software packages by the same (A.L.) and by a different operator (J.M. for Lead-DBS, K.B. for SureTune4™ and BL), respectively. Euclidean distances and differences of θ and φ were calculated. Furthermore, intra- and inter-rater variability of Lead-DBS-delineated VTAs was measured utilizing Dice coefficient that quantifies the similarity of the VTAs from 0 (no match) to 1 (perfect match)^26^ (see Supplementary Fig. 2).

Finally, equivalence analysis (two one-sided T-tests [TOST]) of Euclidean distances for inter-software, inter-rater and intra-rater comparisons was performed, with 2.99 mm empirically considered a clinically significant threshold. Alpha of 0.05 with False Discovery Rate correction^27^ was employed.

## Electronic supplementary material

Below is the link to the electronic supplementary material.

Supplementary Material 1

Supplementary Material 2

## Data Availability

The MRI datasets of the presented study are not publicly available due to data privacy. However, they are available from the corresponding author upon a reasonable request.

## Abbreviations

DBS: Deep brain stimulation
STN: Subthalamic nucleus
T1w: T1-weighted
T2w: T2-weighted
MRI: Magnetic resonance imaging
VTA: volume of tissue activated
ANTs: Advanced normalization tools
AC-PC: Anterior commissure–posterior commissure
MCP: Mid commissural point
LDBS: Lead-DBS
