# Supplementary material for: Precision of post-operative localization of deep brain stimulation electrodes
Source: Sci Rep. 2025 May 28;15:18652. doi: 10.1038/s41598-025-01449-6 (PMC12120071; doi:10.1038/s41598-025-01449-6)

Precision of post-operative localization of deep brain stimulation electrodes

Andrej Lasica MD.[1], Pavel Filip PhD[1,2], Kristína Burdová Mgr. [1], Josef Mana Mgr.[1], Filip Růžička PhD.[1], Dušan Urgošík PhD.[3], Karsten Mueller PhD.[4,1], Dimitra Kiakou[1,4], Robert Jech PhD.[1*]

[1] *Department of Neurology, Charles University, First Faculty of Medicine and General University Hospital, Prague, Czech Republic*

[2] *Center for Magnetic Resonance Research (CMRR), University of Minnesota, Minneapolis, MN, USA*

[3] *Department of Radiology, Na Homolce Hospital, Prague, Czech Republic*

[4] *Max Planck Institute for Human Cognitive and Brain Sciences, Leipzig, Germany*

*** Corresponding author**: Robert Jech, MD, PhD
postal address: Department of Neurology, First Faculty of Medicine
Charles University and General University Hospital in Prague
Kateřinská 30, 120 00 Prague, Czech Republic

Email: jech@cesnet.cz

Supplementary material

Supplementary table 1: MRI acquisition parameters of all pre-operational T1-weighted images used in this study, with the number of subjects where the protocol was utilized (Times used). Abbreviations: IR/GR – Inversion recovery/Gradient recalled, mm – millimeters, T – Tesla, ms – milliseconds, 3D – 3-dimensional

Number of Subjects	Magnetic Field Strength (T)	Manufacturer	MRI scanner type	Acquisition Type	Scanning Sequence	X Resolution	Y Resolution	Z Resolution	Z Dimension (mm)	In Plane Voxel Dimension (mm x mm)	Slice Thickness (mm)	Echo Time (ms)	Repetition Time (ms)	Flip Angle (°)
62	3	SIEMENS	Skyra	3D	IR\GR	224	224	176	1	1.03 x 1.03	1	2.43	2200	8
16	3	SIEMENS	Skyra	3D	IR\GR	256	256	160	1	1 x 1	1	4.43	2300	10
8	3	SIEMENS	TrioTim	3D	IR\GR	256	232	160	1	1 x 1	1	4.43	2300	10
3	3	SIEMENS	TrioTim	3D	IR\GR	256	216	160	1	1 x 1	1	4.43	2300	10
3	3	SIEMENS	TrioTim	3D	IR\GR	256	240	160	1	1 x 1	1	4.43	2300	10
1	1.5	SIEMENS	Avanto	3D	IR\GR	256	208	208	1	1 x 1	1	3.54	2030	15
1	3	SIEMENS	Skyra	3D	IR\GR	256	232	160	1	1 x 1	1	4.43	2300	10
1	3	SIEMENS	Skyra	3D	IR\GR	224	204	176	1	1.03 x 1.03	1	2.43	2200	8
1	3	SIEMENS	Skyra	3D	IR\GR	224	222	176	1	1.03 x 1.03	1	2.43	2200	8
1	3	SIEMENS	Skyra	3D	IR\GR	224	224	167	1	1.03 x 1.03	1	2.43	2200	8
1	1.5	SIEMENS	Symphony	3D	GR	256	224	224	1.5	0.98 x 0.98	1.5	3.08	25	45
1	1.5	SIEMENS	Symphony	3D	GR	256	179	224	1.5	0.98 x 0.98	1.5	3.08	25	45
1	1.5	SIEMENS	Symphony	3D	GR	256	256	160	1.24	0.98 x 0.98	1.24	3.11	25	45
1	1.5	SIEMENS	Symphony	3D	IR\GR	512	512	192	1.17	0.59 x 0.59	1.17	3.93	1870	15
1	3	SIEMENS	TrioTim	3D	IR\GR	256	224	160	1	1 x 1	1	4.43	2300	10
1	3	SIEMENS	TrioTim	3D	IR\GR	256	200	160	1	1 x 1	1	4.43	2300	10
1	3	SIEMENS	TrioTim	3D	IR\GR	256	256	160	1	1 x 1	1	4.43	2300	10
1	3	SIEMENS	TrioTim	3D	IR\GR	256	208	160	1	1 x 1	1	4.43	2300	10

Supplementary table 2: MRI acquisition parameters of all pre-operational T2-weighted images used in this study, with the number of subjects where the protocol was utilized (times used). Abbreviations: SE – Spin echo, mm – millimeters, T – Tesla, ms – milliseconds, 2D – 2-dimensional

Number of subjects	Magnetic Field Strength (T)	Manufacturer	MRI scanner type	Acquisition Type	Scanning Sequence	X Resolution	y Resolution	Z Resolution	Z Dimension (mm)	In Plane Voxel Dimension (mm x mm)	Slice Thickness (mm)	Echo Time (ms)	Repetition Time (ms)	Flip Angle (°)
72	3	SIEMENS	Skyra	2D	SE	256	192	28	2	0.94 x 0.94	2	80	2440	90
6	3	SIEMENS	TrioTim	2D	SE	256	192	28	2	0.94 x 0.94	2	80	2440	90
4	3	SIEMENS	Skyra	2D	SE	256	184	28	2	0.94 x 0.94	2	80	2440	90
4	3	SIEMENS	Skyra	2D	SE	256	176	28	2	0.94 x 0.94	2	80	2440	90
3	3	SIEMENS	TrioTim	2D	SE	256	200	28	2	0.94 x 0.94	2	80	2430	90
3	3	SIEMENS	TrioTim	2D	SE	256	192	28	2	0.94 x 0.94	2	80	2430	90
2	3	SIEMENS	TrioTim	2D	SE	256	176	28	2	0.94 x 0.94	2	80	2440	90
2	3	SIEMENS	TrioTim	2D	SE	256	184	28	2	0.94 x 0.94	2	80	2440	90
1	1.5	SIEMENS	Avanto	2D	SE	256	212	20	2	0.98 x 0.98	2	87	2090	90
1	3	SIEMENS	Skyra	2D	SE	256	200	28	2	0.94 x 0.94	2	80	2440	90
1	3	SIEMENS	Skyra	2D	SE	256	168	28	2	0.94 x 0.94	2	80	2440	90
1	1.5	SIEMENS	Symphony	2D	SE	256	256	18	2	0.8 x 0.8	2	90	2000	90
1	3	SIEMENS	TrioTim	2D	SE	256	180	28	2	0.94 x 0.94	2	80	2440	90
1	3	SIEMENS	TrioTim	2D	SE	256	172	28	2	0.94 x 0.94	2	80	2440	90

Supplementary table 3: MRI acquisition parameters of all post-operational T1-weighted images used in this study, with the number of subjects where the protocol was utilized (times used). Abbreviations: GR/IR – Gradient Recalled/ Inversion Recovery, mm – millimeters, T – Tesla, ms – milliseconds, 3D – 3-dimensional

Number of subjects	Magnetic Field Strength (T)	Manufacturer	MRI scanner type	Acquisition Type	Scanning Sequence	X Resolution	y Resolution	Z Resolution	Z Dimension (mm)	In Plane Voxel Dimension (mm x mm)	Slice Thickness (mm)	Echo Time (ms)	Repetition Time (ms)	Flip Angle (°)
41	1.5	SIEMENS	Symphony	3D	IR\GR	512	512	176	0.9	0.45 x 0.45	0.9	3.93	2060	15
29	1.5	SIEMENS	Avanto	3D	IR\GR	512	512	176	0.9	0.45 x 0.45	0.9	3.31	2060	15
25	1.5	SIEMENS	Symphony	3D	IR\GR	512	512	176	0.9	0.45 x 0.45	0.9	3.93	2150	15
6	3	SIEMENS	Skyra	3D	IR\GR	224	224	176	1	1.03 x 1.03	1	2.43	2200	8
1	1.5	SIEMENS	Avanto	3D	IR\GR	512	512	176	0.9	0.45 x 0.45	0.9	3.31	2130	15
1	1.5	SIEMENS	Symphony	3D	IR\GR	512	480	176	0.9	0.45 x 0.45	0.9	3.93	2060	15
1	1.5	SIEMENS	Symphony	3D	IR\GR	512	512	192	0.9	0.45 x 0.45	0.9	3.93	2150	15
1	1.5	SIEMENS	Symphony	3D	IR\GR	256	256	176	0.9	0.45 x 0.45	0.9	3.93	2060	15

Supplementary table 4: Shows differences in polar angles between the electrode trajectories in each comparison after the coordinates transformation from software packages were reverted. The differences are represented as medians (with 10th-90th percentile).

Comparison type	θ [°]	φ [°]
Lead-DBS vs. SureTune4	15.86 (2.7-42.3)	11.78 (2.97-19.62)
Lead-DBS vs. BrainLab Elements	1.59 (0.27-5.28)	1.35 (0.02-3.19)
SureTune4 vs. BrainLab Elements	15.24 (3.23-40.96)	11.11 (2.7-20.27)
Lead-DBS intra-rater	1.37 (0.31-6.09)	1.0 (0.084-2.21)
Lead-DBS inter-rater	2.97 (0.54-6.76)	1.46 (0.22-3.39)
SureTune4 intra-rater	1.64 (0.24-4.85)	1.03 (0.3-2.58)
SureTune4 inter-rater	2.74 (0.37-7.65)	3.33 (0.87-8.3)
BrainLab Elements intra-rater	1.21 (0.22-2.88)	0.69 (0.11-1.57)
BrainLab Elements inter-rater	1.21 (0.21-3.59)	0.53 (0.13-2.19)

Supplementary figure 1: Depiction of polar angles θ and φ. Axes x, y, and z are defined by the MRI system settings. Position vector of the path of the lead is calculated after moving the path so that the first contact of the lead coincides with the reference point (0,0,0). Point A is the proximal contact of the electrode. θ is the angle between point C as the projection of the point A onto the x-y plane and the positive part of the x axis, with vertex in the zero reference point. φ is then defined as the angle between point C and point A, with vertex in the zero reference point.

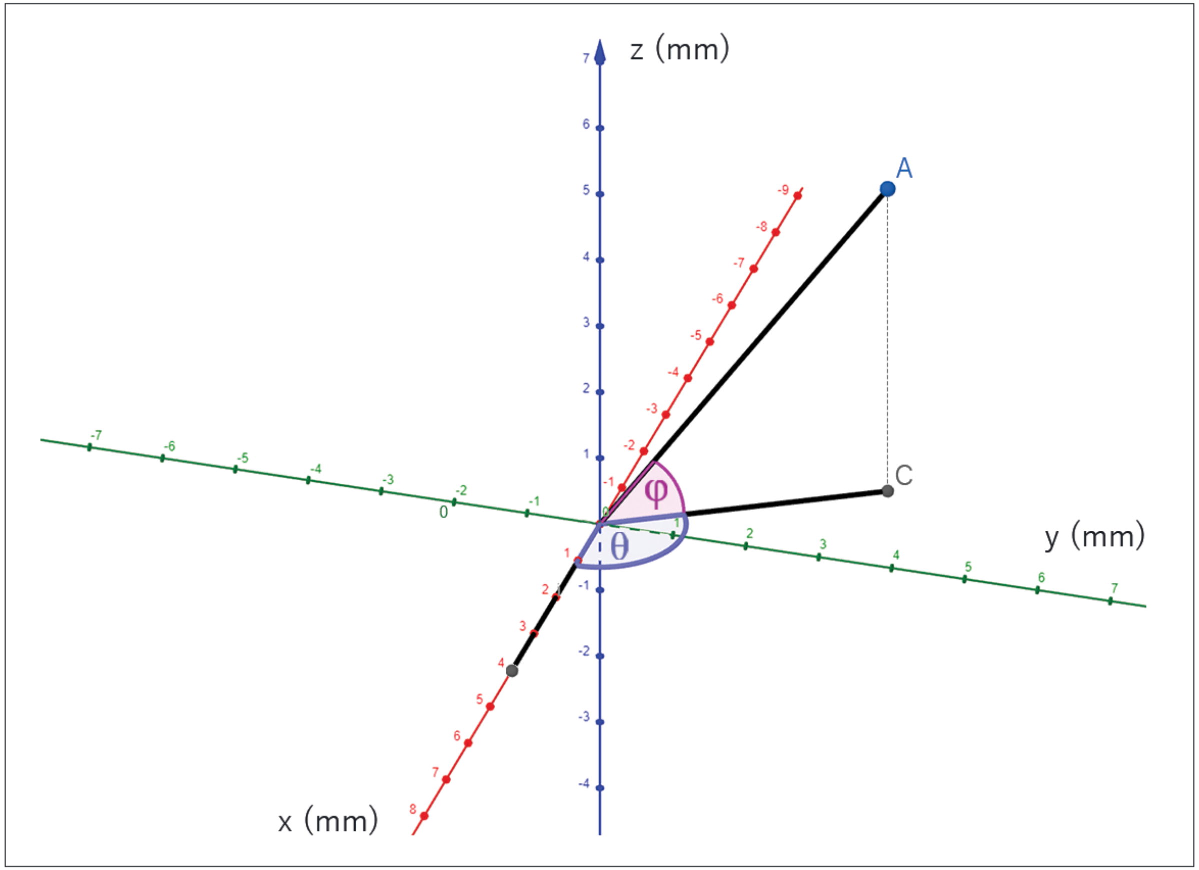

Supplementary figure 2: Visualization of the calculation of Dice coefficient for Volume of Tissue Activated (VTA) in Lead-DBS software; intra-rater evaluation of a representative subject with Dice coefficient of 0.779, close to the median of the dataset; in transversal view. The green color represents the part of VTA as the output of the first processing run that does not intersect with the VTA from the second processing run. Conversely, purple marks the part of VTA from the second processing run that does not intersect with the first run. Finally, the red color represents the intersection of the two processing runs.

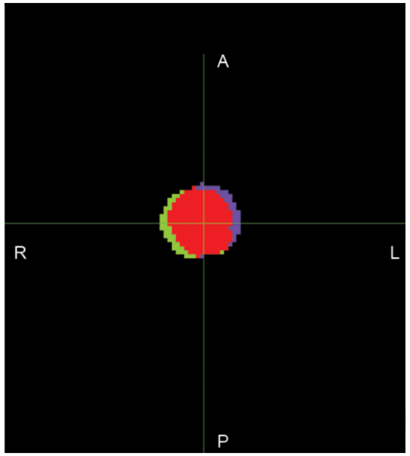

Supplementary figure 3: Violin graphs for inter-software package comparisons after reversion of Lead-DBS and BrainLab Elements transformations. Medians of Euclidean distances between positions of active contacts estimated by individual software packages are marked with a black dot, with 95% confidence intervals shown with the error bars. Dashed black line marks the width of the subthalamic nucleus for reference as the predetermined threshold of clinical significance. BrainLab Elements software is shortened to "BrainLab" because of space issues.

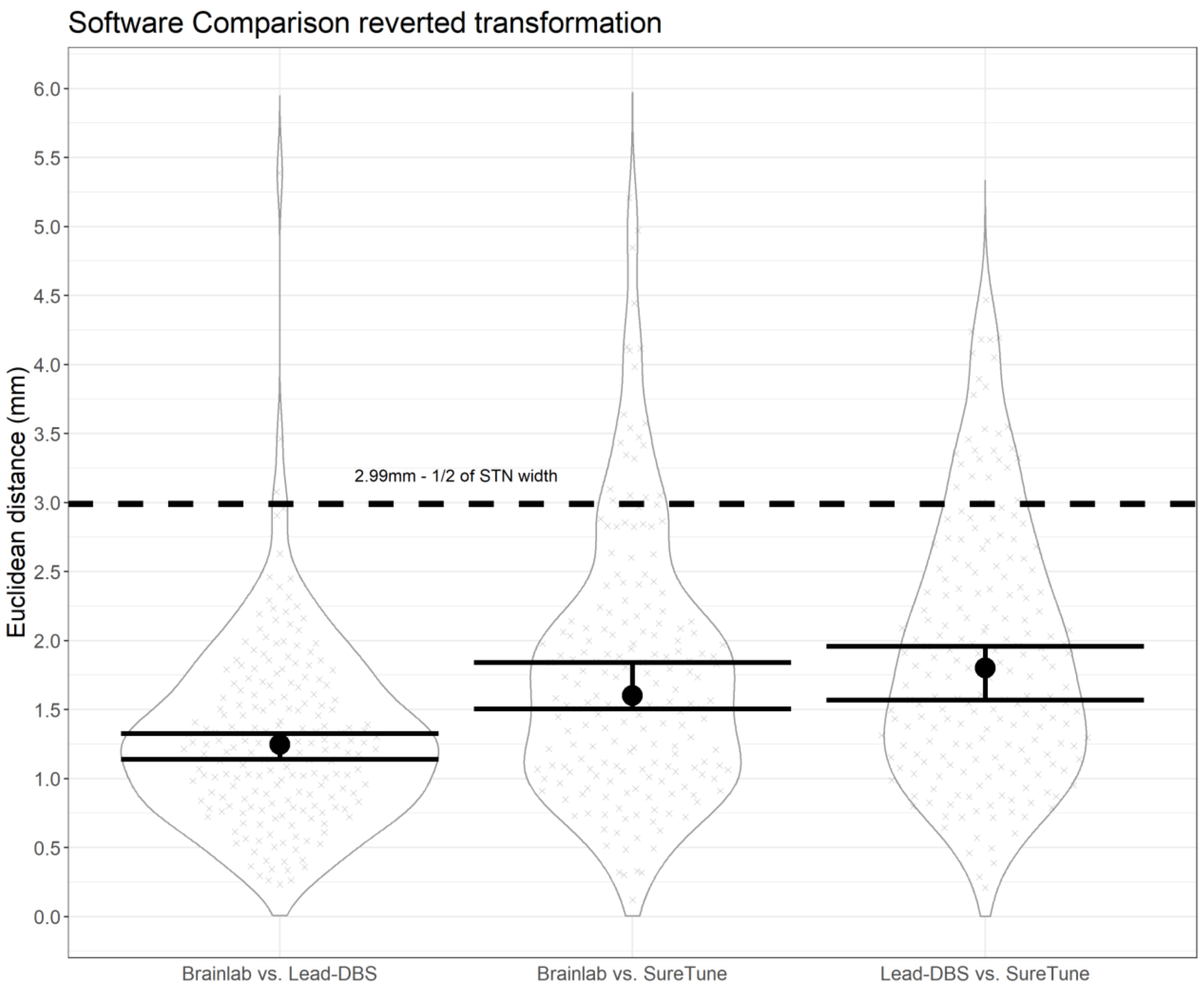

Supplementary figure 4: Violin graphs for within-software package inter- (A) and intra-rater (B) comparisons separately in individual software packages after the transformations from Lead-DBS and BrainLab Elements were reverted. Medians of Euclidean distances between positions of active contacts estimated by individual software packages are marked with a black dot, with 95% confidence intervals shown with the error bars. The dashed black line marks the width of the subthalamic nucleus for reference as the predetermined threshold of clinical significance.

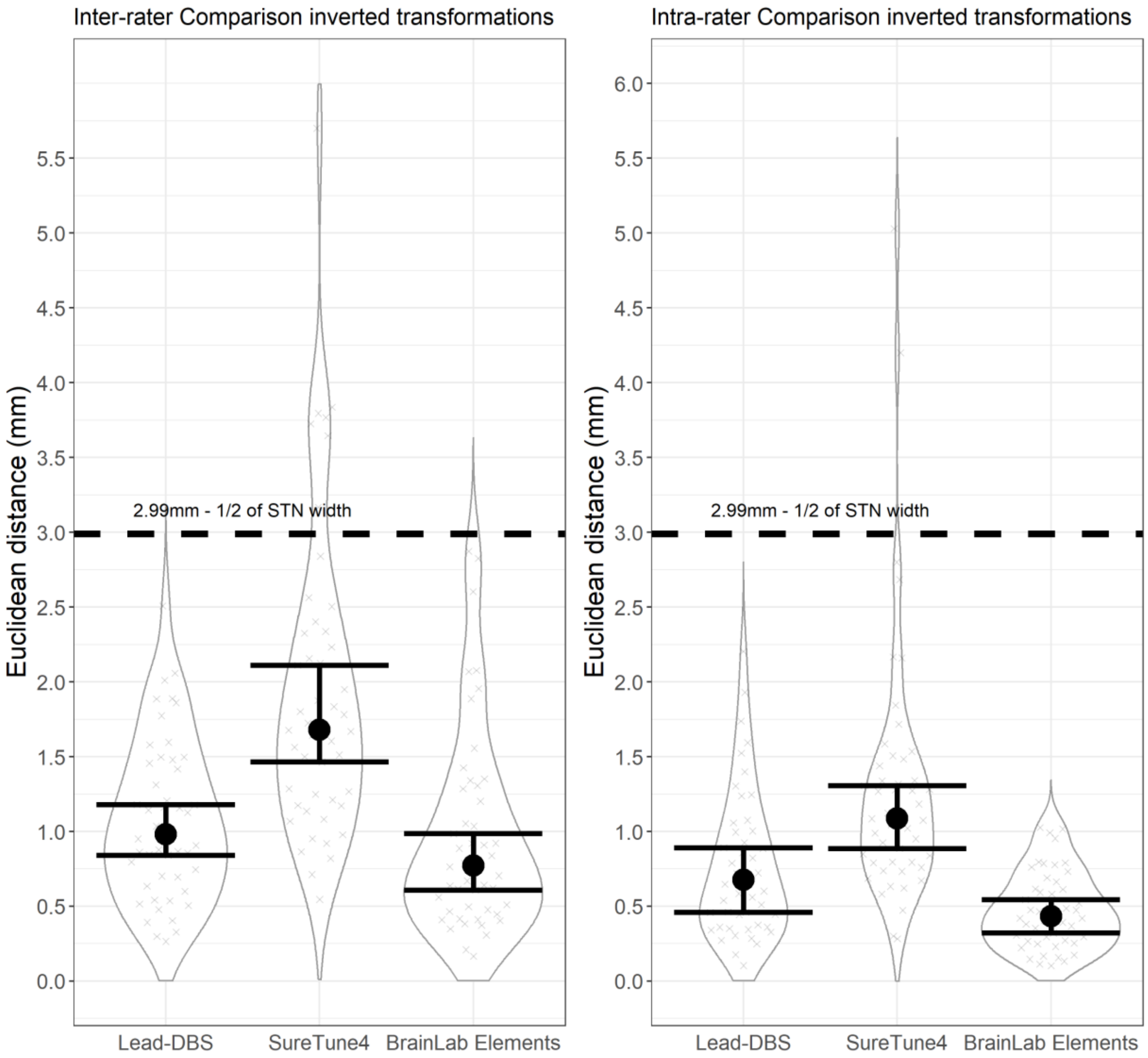

Supplementary figure 5: Differences in individual axes in both software comparison and inter-rater and intra-rater differences. The transformation done by Lead-DBS and BrainLab Elements were reverted before the analysis of axial differences. Axis x shows the axial difference in mm and y axis shows the analysis and the analyzed axis. Medians of axial differences are represented by black dot, with 95% confidence intervals shown with the error bars.

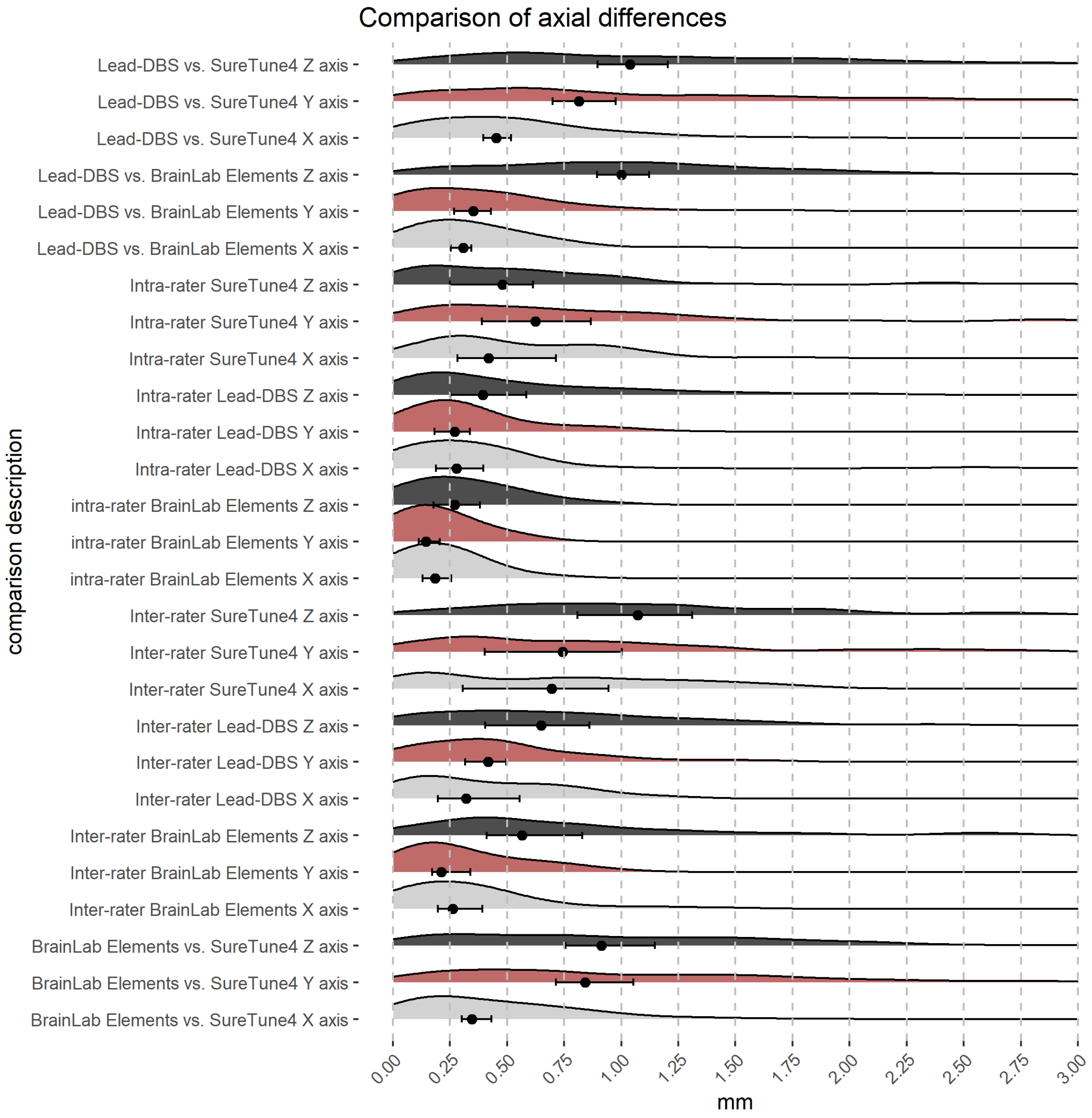

Supplementary figure 6: Violin graphs for the closest distance between the bejjani line passing through the anterior part of both Red Nuclei and the active contact in each software separately. Medians of closest distances between positions of active contacts and the closest point on the bejjani line estimated by individual software packages are marked with a black dot, with 95% confidence intervals shown with the error bars. The transformation of the coordinates used in this analysis were reverted before they were used in this analysis.

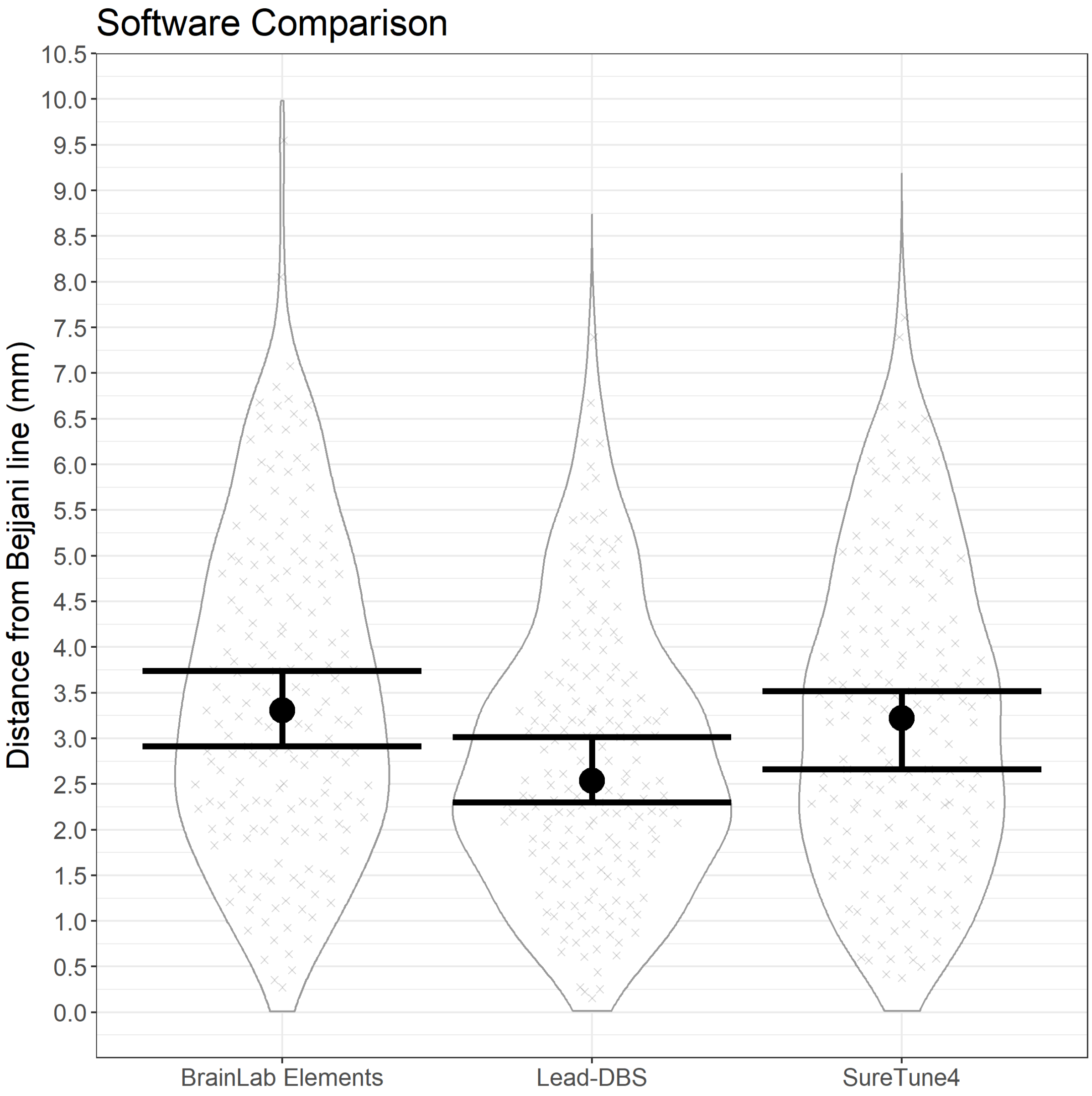

Supplementary figure 7: Violin graphs for within-software package second try of the same rater (A) and different rater (B) Violin graphs for the closest distance between the bejjani line passing through the anterior part of both Red Nuclei and the active contact in each software separately. Medians of closest distances between positions of active contacts and the closest point on the bejjani line estimated by individual software packages are marked with a black dot, with 95% confidence intervals shown with the error bars. The transformation of the coordinates used in this analysis were reverted before they were used in this analysis. BrainLab Elements software is shortened to "BrainLab" because of space issues.

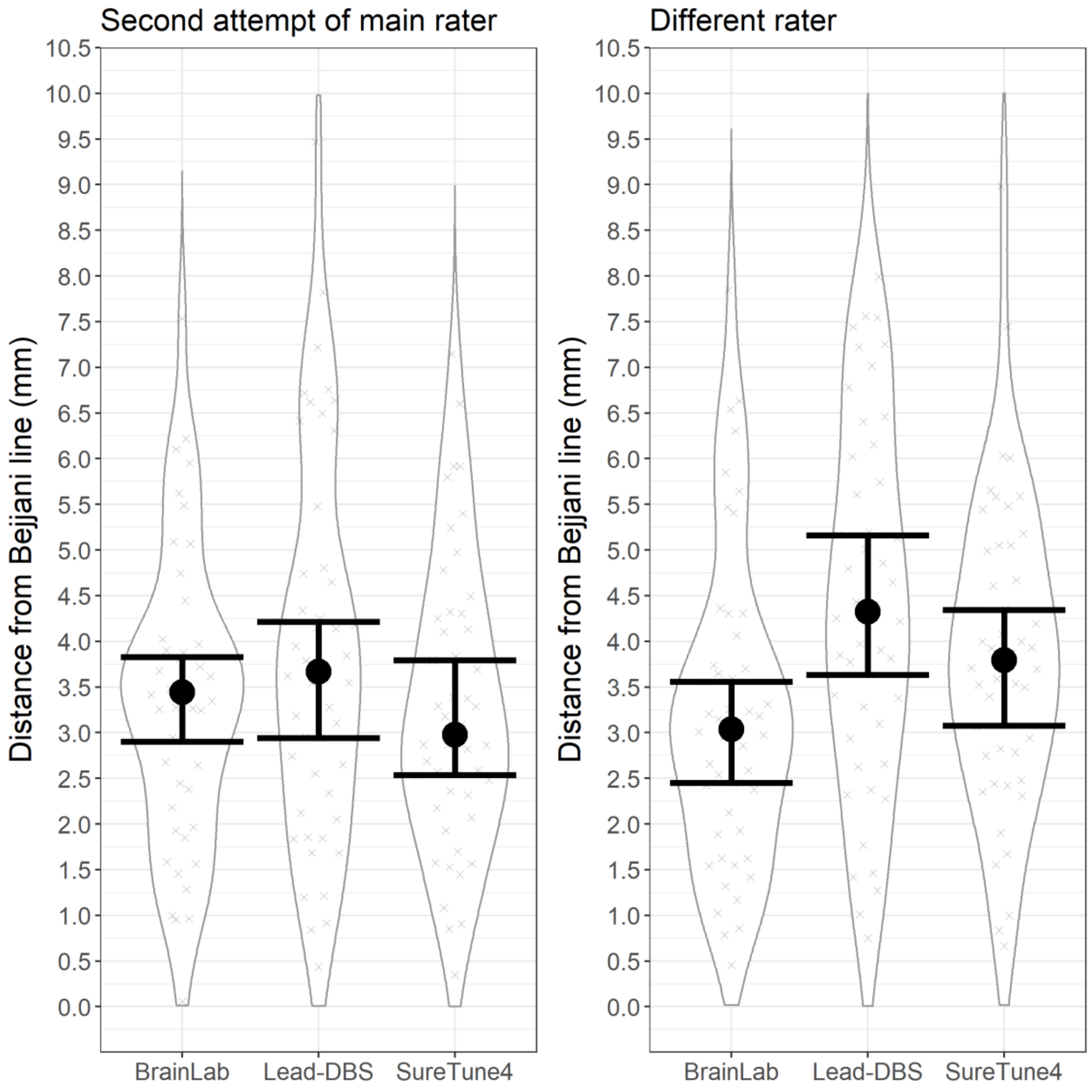

Supplement: Supplementary file 2 — Supplementary Material 2 [file 41598_2025_1449_MOESM2_ESM.pdf]
